# Protection Effect of Endomorphins on Advanced Glycation End Products Induced Injury in Endothelial Cells

**DOI:** 10.1155/2013/105780

**Published:** 2013-04-08

**Authors:** Jing Liu, Liping Yan, Ruilan Niu, Limin Tian, Qi Zhang, Jinxing Quan, Hua Liu, Suhong Wei, Qian Guo

**Affiliations:** ^1^Department of Endocrinology, Gansu Provincial People's Hospital, 204 West Donggang Road, Lanzhou City 730000, Gansu Province, China; ^2^The First Clinical College of Lanzhou University, Lanzhou City 730000, Gansu Province, China; ^3^Department of Pneumology, Gansu Provincial People's Hospital, 204 West Donggang Road, Lanzhou City 730000, Gansu Province, China

## Abstract

Endomorphins (EMs) have a very important bridge-function in cardiovascular, endocrinological, and neurological systems. This study is to investigate the effects of EMs on the synthesis and secretion of vasoactive substances induced by advanced glycation end products in primary cultured human umbilical vein endothelial cells (HUVECs). Firstly, HUVECs were stimulated with AGEs-bovine serum albumin (AGEs-BSA), bovine serum albumin (BSA), or both AGEs-BSA and EMs together, respectively. Then, HUVEC survival rate was calculated by MTT assay, the levels of NO, endothelial nitric oxide synthase (eNOS), and inducible nitric oxide synthase (iNOS) were detected by colorimetric analysis, and the contents of endothelin-1 (ET-1) were detected by ELISA. The mRNA levels of eNOS and ET-1 were measured by RT-PCR. The expression of p38 mitogen-activated protein kinase (p38 MAPK) was detected by immunofluorescence assay. The results showed that the mRNA expression and secretion of eNOS were significantly enhanced after incubation with EMs compared to those with AGEs-BSA, while the secretion of NO and iNOS, mRNA expression, and secretion of ET-1 had opposite changes. The fluorescence intensity of p38MAPK in nuclear was decreased after pretreatment with EMs compared to incubation with AGEs-BSA. *Conclusion*. The present study suggests that EMs have certain protection effect on AGEs-BSA-induced injury in HUVEC.

## 1. Introduction

Overwhelming evidence proved that the formation and accumulation of advanced glycation end products (AGEs) progress in a normal aging process and at an accelerated rate under diabetes [1, 2]; an increase in the steady-state levels of highly reactive dicarbonylic compounds may lead to the formation of AGEs, while an increase in the generation of AGEs can be partly explained by the process of non-enzymatic glycosylation of proteins. These proteins appear to contribute to diverse cellular functions, such as the specific recognition and degradation of AGEs-modified proteins [3]. So far, several AGE-binding proteins have been identified, including AGE-R1, AGE-R2, AGE-R3, RAGE, and macrophage scavenger receptors type I and type II. In endothelial cells, AGEs exert adverse effects on mitochondrial function, with elevated production of reactive oxygen species (ROS), and consequently increased oxidative stress leading to cellular dysfunction and even cell death. AGEs also increase the formation of intracellular ROS, NO, and nitric oxide synthase (NOS) and stimulate ceramides as well as the MAPK cascade, which activates different targets including transcription factors through intermediate molecules such as NF-*κ*B [4–6]. Therefore preventing the endothelial cell from AGE-triggered injury may improve diabetes-associated vascular complications.

The endogenous opioid peptides, endomorphin 1 (Tyr1-Pro2-Trp3-Phe4-NH2, EM1) and endomorphin 2 (Tyr1-Pro2-Phe3-Phe4-NH2, EM2), which were discovered in 1997 by Champion et al., have higher affinity and are more selective for the *μ*-opiate receptor than other opioid substances [7]. Many studies indicated that the endogenous opioid system played roles in the regulation of the cardiovascular system in a variety of species [8, 9], such as rabbits [10], rats [11, 12], and mice [13] Furthermore, Jaffe et al. [14] reported that vasodilator responses to endomorphin 1 were mediated by a nitric oxide-dependent mechanism and may act as an endothelium-dependent vasodilator agent in rat. However, the precise molecular mechanisms by which EMs inhibit AGE-induced injury in endothelial cells have not yet been thoroughly elucidated. The purpose of this study is to investigate the inhibitory effects and to involve mechanisms of EMs on AGEs induced-oxidative stress and apoptosis in human umbilical vein endothelial cells. 

## 2. Materials and Methods

### 2.1. Reagents

Endomorphins was synthesized by Shanghai Hanhong Chemical Co., Ltd (Shanghai, China). Fetal bovine serum (FBS) was obtained from Hangzhou Sijiqing Biological Engineering Materials (Hangzhou, China). NO and endothelial nitric oxide synthase (eNOS) assay kits were obtained from Jian-Cheng Biological Engineering Institute (Nanjing, China). Rabbit anti-human P38 (H174) antibody, FITC-conjugated goat anti-rabbit antibody were obtained from Bioworld Technology, Inc. (Minneapolis, USA). BSA was purchased from Sigma (St. Louis, MO, USA). The primers, Taq polymerase, dNTP, and Rnasin were provided by TaKaRa Bio Inc. (Otsu, Shiga, Japan).

### 2.2. Preparation of AGEs

AGEs-BSA was produced by incubation of 10 mg/mL BSA with 100 mM glucose in 150 mM phosphate-buffered saline (PBS), pH 7.4 at 37°C for 6 weeks [15]. Control BSA was incubated in the same conditions without glucose. Unbound sugar was removed by centrifugation filtration with Centricon filter cartridges. AGEs-BSA was identified by fluorescence spectrophotometer.

### 2.3. Cell Culture and Treatment

Before the study, we recruited mothers who assented and gave written consent to contributing 10 cm of umbilical cord postpartum, and were isolated according to a previous reported method [16] with minor modifications. Cultured cells were identified as endothelial by their morphology and the presence of von Willebrand factor. Briefly, the cells were grown in DMEM supplemented with 10% fetal bovine serum, penicillin (100 units/mL), and streptomycin (100 mg/mL). The cultures were maintained at 37°C in a humidified atmosphere of 5% CO_2_. Culture medium was refreshed every two days. For experiments, cells treated with endomorphins (10 *μ*M, 1 *μ*M, 0.1 *μ*M, or 10 nM) were exposed to these substances for 2 h before treatment with AGEs-BSA.

### 2.4. Cell Viability Assay

Cells were incubated in 96-well plates at a density of 5 × 10^3^ cells with 200 *μ*L culture medium per well. After cells were incubated according to the aforementioned group, 30 *μ*L medium containing 5 mg/mL MTT (Sigma, USA) was added to each well. Following a 4 h incubation period, 100 *μ*L 10% SDS was added. And then, after overnight incubation in darkness, the dissolved MTT crystals were quantified. Optical densities were obtained using a test at a wavelength of 570 nm.

### 2.5. Chemiluminescence Analysis of NO

Levels of the nitric oxide (NO) derivative nitrite were determined in the conditioned medium of HUVEC with the Griess reaction [17]. After cells were incubated according to the aforementioned grouping, 100 *μ*L culture solutions of each well was collected and put into the counterpart well of another plates, then NO production in cells was measured by Griess method and according to the indication on the NO assay kit. Optical density was read in a microplate reader at 540 nm. Each experiment was performed in triplicate.

### 2.6. Determination of eNOS and iNOS Activity

After cells were incubated according to the aforementioned grouping, 200 *μ*L culture solutions of each well was collected and put into the counterpart well of another plates, then eNOS and iNOS expression in cells were measured according to the instructions given in the NOS assay kit. Optical density was read in a microplate reader at 530 nm. Each experiment was performed in triplicate.

### 2.7. ELISA Analysis of Endothelin-1

A specific sandwich enzyme-linked immunosorbent (ELISA) employing monoclonal antibody was used to determinate the level of ET-1; the ELISA was performed according to the instructions given in the ET-1 ELISA kit by Ad Litteram Diagnostic Laboratories (USA). Optical density was read in a microplate reader at 450 nm. Each experiment was performed in triplicate.

### 2.8. Real-Time RT-PCR Analysis for eNOS and ET-1 mRNA Level

After incubation, the cells were washed twice with PBS and the total mRNA was extracted by Trizol. Thereafter, it was reverse-transcripted under following conditions: 37°C for 15 min, 85°C for 5 sec, and the cDNA product was stored at −80°C. For the PCR, 3 *μ*L of the cDNA products of each sample was amplified with Taq DNA polymerase, using a primer pair specific to human eNOS, ET-1, and *β*-actin in a 25 *μ*L reaction volume; the primer sequences and PCR condition were described in Table 1. PCR cycle conditions were 95°C for 30 sec, 95°C for 5 sec, 60°C for 30 sec for 50 cycles, with an initial denaturation at 94°C for 5 min and a final extension of 5 min at 72°C. The resulting data were analyzed by Rotor-Gene Real-Time analysis software 6.1. The relative mRNA expression level of each targeted gene was calculated by 2^−ΔΔCt^. 

### 2.9. Immunofluorescence Staining

Immunofluorescence staining was performed as described previously [18]. Cells were fixed with 4% paraformaldehyde (pH 7.4) for 15 min at 4°C and permeabilized with 0.2% Triton X-100 for 5 min at room temperature. After being blocked with 5% normal bovine serum for 30 min, cells were incubated with p38 MAPK antibody (1 : 100 dilution) at 4°C overnight followed by FITC-conjugated secondary antibody (1 : 50 dilution, 1 h). Images were obtained using fluorescence microscope (IX81, Olympus, Japan).

### 2.10. Statistical Analysis

Statistical evaluations were performed using one-way ANOVA followed by Tukey's test. Values of *P* < 0.05 were considered statistically significant. Data are expressed as mean ± SE of at least three independent experiments.

## 3. Results

### 3.1. Effect of EMs on Cell Viability

**Figure 1:**
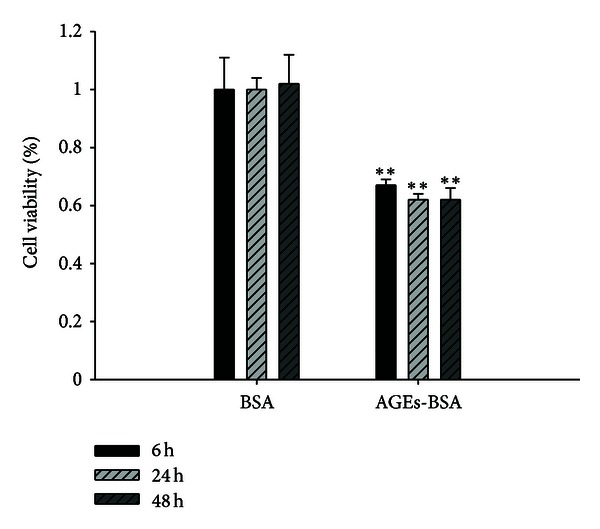
Effect of AGEs-BSA on cell viability determined by MTT test. HUVECs were treated with AGEs-BSA (100 mg/L) or BSA (100 mg/L) for 6 h, 24 h, 48 h. Viability was calculated as the percentage of living cells in treated cultures compared to those in control cultures. Each value represents the mean ± SD (*n* = 3). *P < 0.05,  **P < 0.01 versus BSA group.

**Figure 2:**
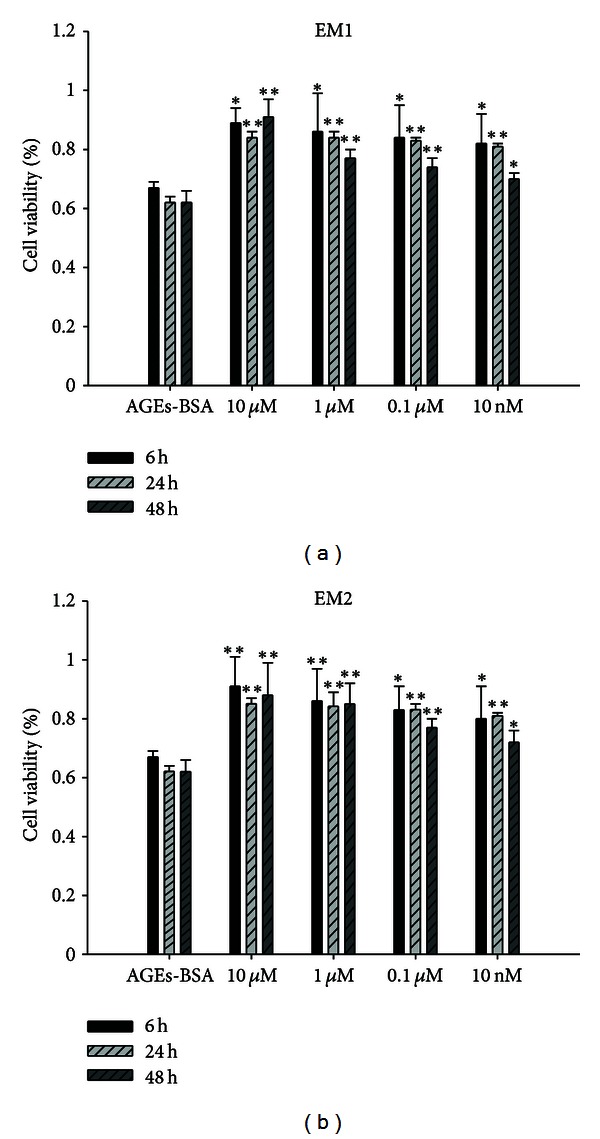
Effect of EM1, EM2 on cell viability determined by MTT test. HUVECs were treated with EMs (10 *μ*M, 1 *μ*M, 0.1 *μ*M, 10 nM) for 2 h before treatment with AGEs-BSA (100 mg/L) for 6 h, 24 h, 48 h. Viability was calculated as the percentage of living cells in treated cultures compared to those in control cultures. Each value represents the mean ± SD (*n* = 3). Statistical analysis compared with AGEs-BSA group by ANOVA. *P < 0.05,  **P < 0.01.

Exposure of HUVEC to AGEs-BSA (100 mg/L) for 6 h, 24 h, and 48 h significantly decreased the cells viability significantly compared to that of BSA (100 mg/L, as osmotic control) (*P* < 0.01, Figure 1). The cell viability was decreased at 6 h and reached minimal level at 48 h after AGEs-BSA treatment. Whereas pretreatment with EM1 and EM2 (10 *μ*M, 1 *μ*M, 0.1 *μ*M, 10 nM) significantly increased the cells viability compared to AGEs-BSA group, the function was obvious at 24 h, 48 h compared to 6 h (*P* < 0.005 versus *P* < 0.05), and high concentration was more obvious than low concentration, which indicated that EMs can attenuate the reduction of cell viability by AGEs-BSA in a time- and concentration-dependent manner (Figure 2). 

### 3.2. Effect of EMs on NO Production

**Figure 3:**
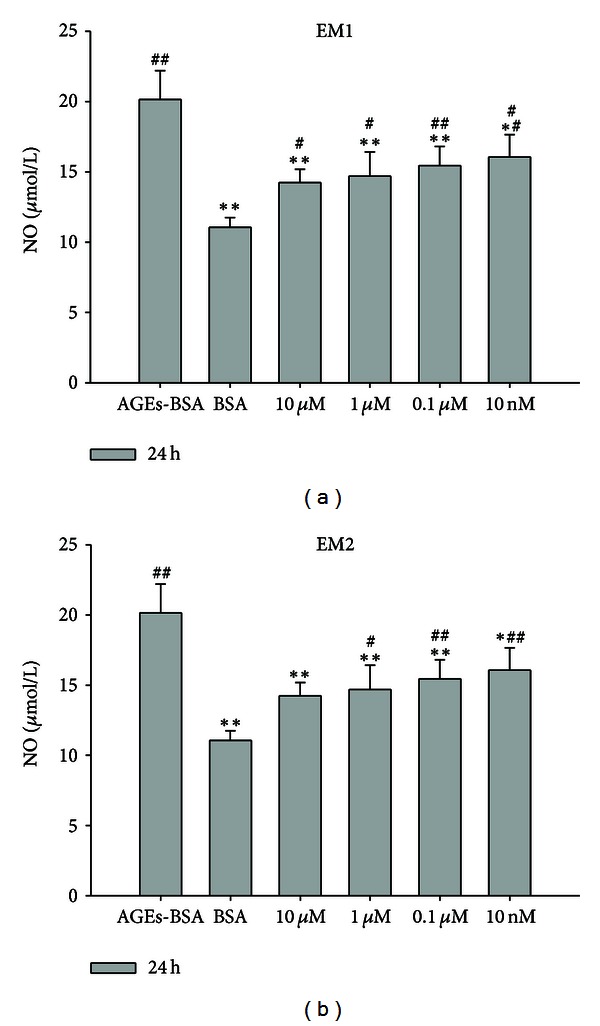
Effect of EM1, EM2 on NO concentration determined by Griess reaction test in HUVEC. Each data is expressed as mean ± SD (*n* = 3). *P < 0.05,  **P < 0.005 versus AGEs-BSA group, ^#^ *P* < 0.05,  ^##^ *P* < 0.005 versus control (BSA) group.

As is shown in Figure 3, the NO production in HUVEC was 11.06 ± 0.69 *μ*M after incubation for 24 h in the control group and was 20.15 ± 2.05 *μ*M in the AGEs-BSA group, which was notably higher than that of control group (*P* < 0.005), while the NO production in HUVEC were 14.24 ± 0.95 *μ*M, 14.70 ± 1.72 *μ*M, 15.45 ± 1.36 *μ*M, 16.06 ± 1.60 *μ*M after incubation for 24 h in EM1 pretreated group as the concentrations of 10 *μ*M, 1 *μ*M, 0.1 *μ*M, 10 nM, which were notably lower than that of AGEs-BSA group (*P* < 0.005, 0.05). These results indicated that EM1 inhibited the NO production in a concentration-dependent manner in HUVEC stimulated by AGEs-BSA. The same results were observed in EM2 group.

### 3.3. Effect of EMs on iNOS Secretion

**Figure 4:**
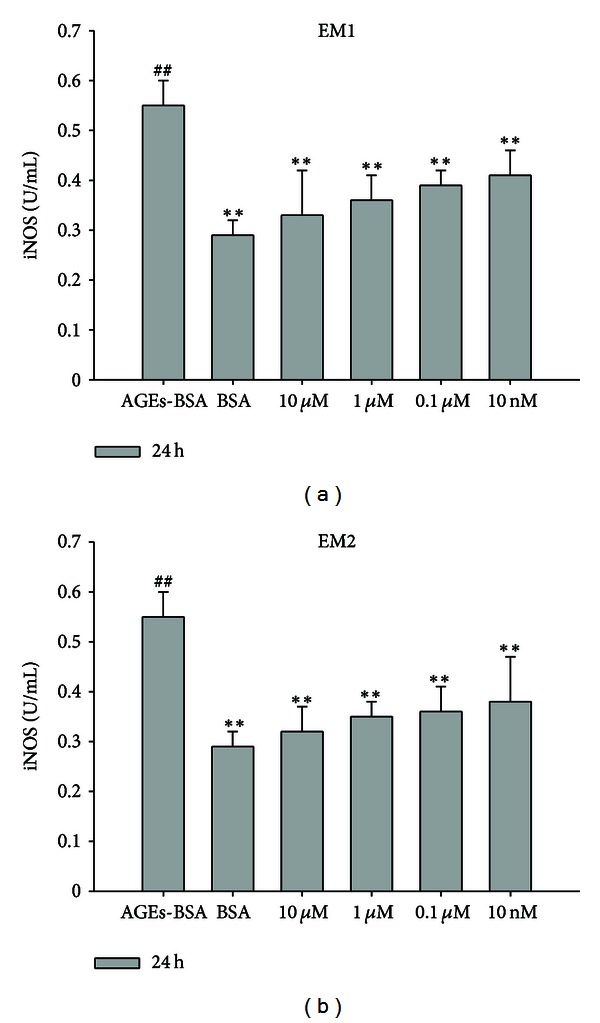
Effect of EM1, EM2 on iNOS level in HUVEC. Each data is expressed as mean ± SD (*n* = 3). *P < 0.05,  **P < 0.005 versus AGEs-BSA group, ^#^ *P* < 0.05,  ^##^ *P* < 0.005 versus control (BSA) group.

In the control group (see Figure 4), the secretion of iNOS was 0.29 ± 0.03 U/mL after incubation for 24 h and was 0.55 ± 0.05 U/mL in AGEs-BSA treated group, which was significantly increased to the control group (*P* < 0.005). While the iNOS secretion in EM1, EM2 pretreated groups were 0.33 ± 0.09 U/mL, 0.36 ± 0.05 U/mL, 0.39 ± 0.03 U/mL, 0.41 ± 0.05 U/mL; 0.32 ± 0.05 U/mL, 0.35 ± 0.03 U/mL, 0.36 ± 0.05 U/mL, 0.38 ± 0.09 U/mL as the concentrations of 10 *μ*M, 1 *μ*M, 0.1 *μ*M, 10 nM, which were significantly decreased to the AGEs-BSA treated group (*P* < 0.005, 0.05), these results indicated that EMs efficiently and concentration-dependently inhibited the iNOS secretion in HUVEC.

### 3.4. Effect of EMs on eNOS Secretion, mRNA Level of eNOS

**Figure 5:**
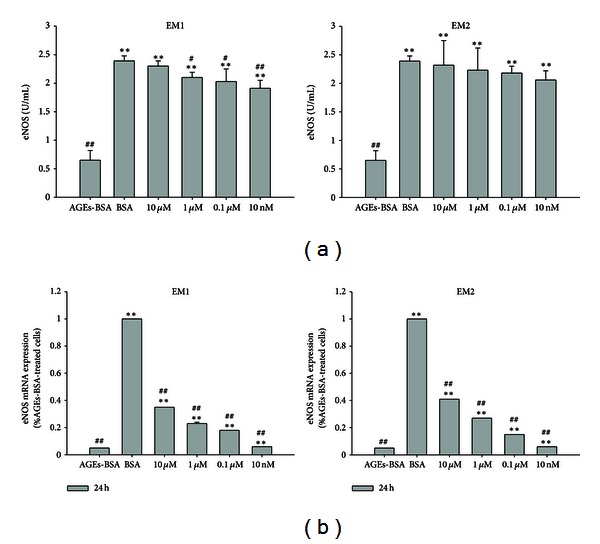
Effect of EM1, EM2 on eNOS secretion determined by ELISA test in HUVEC (a). HUVECs were incubated according to the aforementioned grouping. Each value represents the mean ± SD (*n* = 3); mRNA expression level of eNOS after treatment with AGEs-BSA and EM1 or EM2, using BSA treated cells as reference control (b). The parameter Ct was derived for each cDNA sample and primer pair; for a given sample, Ct values for *β*-actin were subtracted from the Ct of each candidate gene reaction to arrive at a ΔCt value. The mean ΔCt from all control reactions was then subtracted from the ΔCt of each treated sample to arrive at ΔΔCt. The relative fold change was calculated by the expression 2^−ΔΔCt^. Each data is expressed as mean ± SD (*n* = 3). *P < 0.05,  **P < 0.005 versus AGEs-BSA group, ^#^ *P* < 0.05,  ^##^ *P* < 0.005 versus control (BSA) group.

In the control group (see Figure 5(a)), the secretion of eNOS was 2.39 ± 0.09 U/mL after incubated for 24 h, and that of AGEs-BSA treated group was 0.65 ± 0.17 U/mL in, which was significantly decreased compared to the control group (*P* < 0.005). While the secretions of eNOS in EM1, EM2 pretreated groups were 2.30 ± 0.09 U/mL, 2.10 ± 0.09 U/mL, 2.03 ± 0.22 U/mL, 1.91 ± 0.14 U/mL; 2.32 ± 0.43 U/mL, 2.23 ± 0.39 U/mL, 2.18 ± 0.12 U/mL, 2.06 ± 0.16 U/mL as the concentrations of 10 *μ*M, 1 *μ*M, 0.1 *μ*M, 10 nM, which were significantly higher compared to the AGEs-BSA treated group (*P* < 0.005, 0.05), these results indicate that EMs pretreatment abrogated the decrease efficiently, and in a concentration-dependent manner. Similar results were observed for the mRNA level of eNOS (Figure 5(b)). These results indicate that EMs efficiently inhibited the decrease of eNOS expression and secretion stimulated by AGEs in HUVEC.

### 3.5. Effect of EMs on ET-1, mRNA Level of ET-1

**Figure 6:**
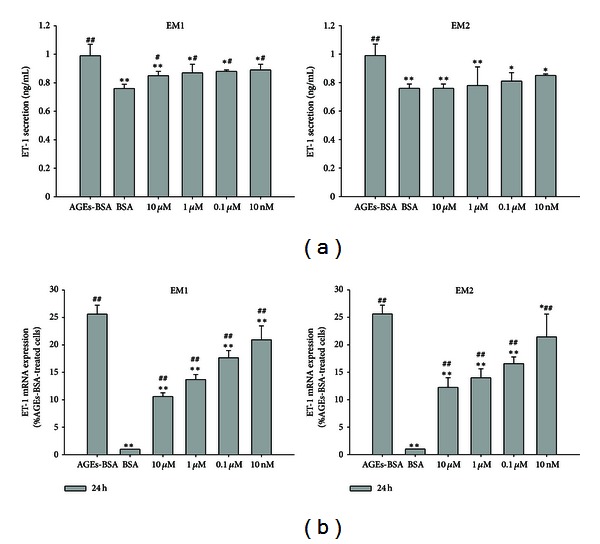
Effect of EM1, EM2 on ET-1 secretion determined by ELISA test in HUVEC (a). HUVECs were incubated according to the aforementioned grouping. Each value represents the mean ± SD (*n* = 3); mRNA expression level of ET-1 after treatment with AGEs-BSA and EM1 or EM2, using BSA treated cells as reference control (b). The parameter Ct was derived for each cDNA sample and primer pair; for a given sample, Ct values for *β*-actin were subtracted from the Ct of each candidate gene reaction to arrive at a ΔCt value. The mean ΔCt from all control reactions was then subtracted from the ΔCt of each treated sample to arrive at ΔΔCt. The relative fold change was calculated by the expression 2^−ΔΔCt^. Each data is expressed as mean ± SD (*n* = 3). *P < 0.05,  **P < 0.005 versus AGEs-BSA group, ^#^ *P* < 0.05,  ^##^ *P* < 0.005 versus control (BSA) group.

In the control group (Figure 6(a)), the secretion of ET-1 was 0.76 ± 0.03 ng/mL after incubated for 24 h and was 0.99 ± 0.08 ng/mL in AGEs-BSA treated group, which was significantly higher than that in the control group (*P* < 0.005). While the ET-1 secretions in EM1, EM2 pretreated groups were 0.85 ± 0.03 ng/mL, 0.87 ± 0.06 ng/mL, 0.88 ± 0.01 ng/mL, 0.89 ± 0.04 ng/mL; 0.76 ± 0.03 ng/mL, 0.78 ± 0.13 ng/mL, 0.81 ± 0.06 ng/mL, 0.85 ± 0.01 ng/mL as the concentrations of 10 *μ*M, 1 *μ*M, 0.1 *μ*M, 10 nM, which were significantly lower than the AGEs-BSA treated group (*P* < 0.005, 0.05), these results indicated that EMs pretreatment abrogated the increase efficiently in a concentration-dependent manner. Similar results were observed for the mRNA level of ET-1 (Figure 6(b)). These results indicated that EMs efficiently inhibited the ET-1 mRNA expression and ET-1 secretion in HUVEC.

### 3.6. Effect of EMs on p38 MAPK

**Figure 7:**
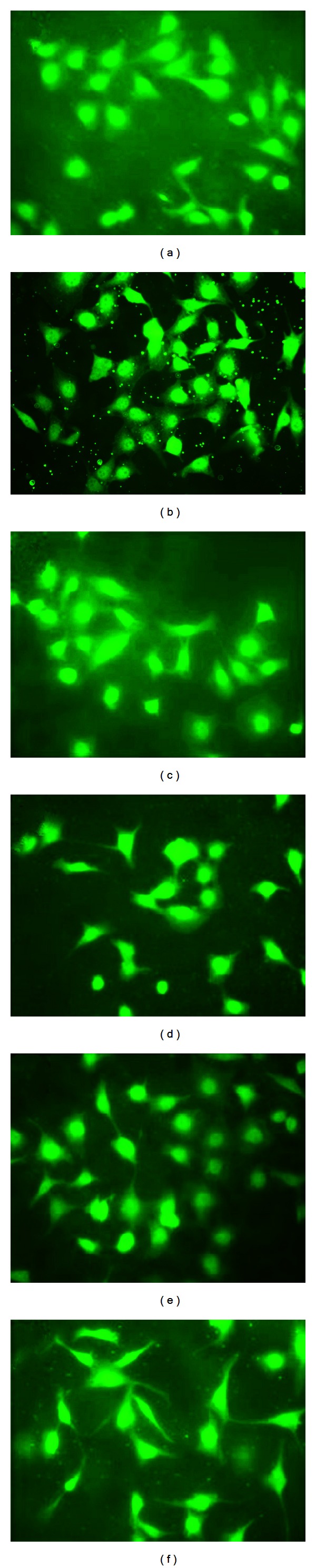
Immunofluorescence studies of EM1 on p38 MAPK in HUVECs. Cells were fixed, and incubated with p38 MAPK antibody and a FITC-conjugated second antibody. Pictures were taken at 400x magnification. ((a) BSA, (b) AGEs-BSA, (c) 10 *μ*M, (d) 1 *μ*M, (e) 0.1 *μ*M, (f) 10 nM).

**Figure 8:**
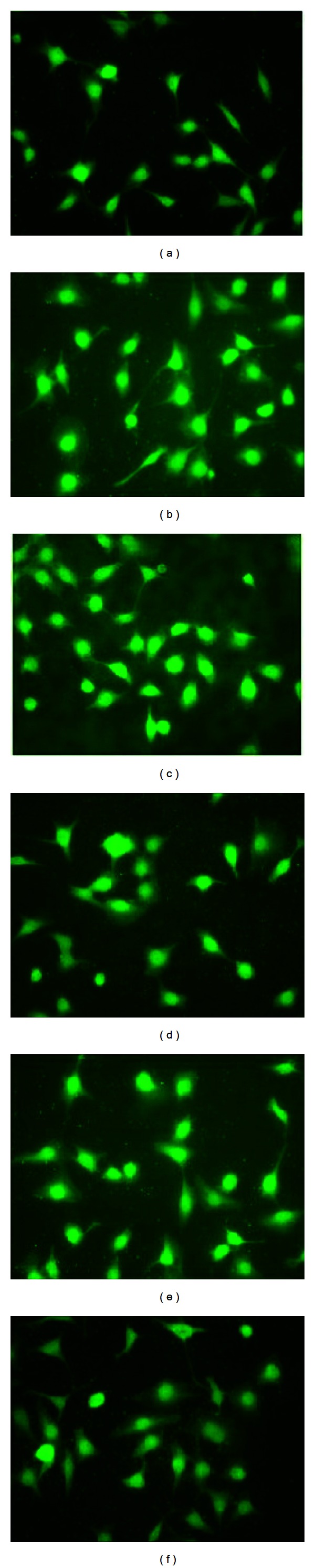
Immunofluorescence studies of EM2 on p38 MAPK in HUVECs. Cells were fixed and incubated with p38 MAPK antibody and a FITC-conjugated second antibody. Pictures were taken at 400x magnification. ((a) AGEs-BSA, (b) BSA, (c) 10 *μ*M, (d) 1 *μ*M, (e) 0.1 *μ*M, (f) 10 nM).

In this study, our investigation tries to ascertain whether EMs inhibit the AGEs-induced dysfunction in endothelial cells through p38 MAPK activities. As noted in Figures 7(b) and 8(a), the fluorescence intensity of p38 MAPK in the nucleus was obviously elevated in AGEs-treated HUVECs relative to that in BSA-treated group (Figures 7(a) and 8(b)). However, in EMs pretreated groups (Figures 7(c)–7(f)) and Figures 8(c)–8(f)), the fluorescence intensity of p38 MAPK in the nucleus was similar to BSA group, obviously weaker compared to AGEs-BSA group. Therefore, these results implied that EMs inhibited the expression of p38 MAPK in the nucleus induced by AGEs.

## 4. Discussion

Vascular endothelial cells play an important role in modulating anti-thrombus and maintaining the natural function of vascular by secreting many active substances. AGEs, high blood glucose, oxide-LDL, and inflammatory factor are the main factors that induce endothelial cells injury [19, 20]. Once endothelial cell were damaged, it would result in dysfunction and abnormal secretion of active substances (e.g. NO, NOS, ET-1, and prostacyclin PGI_2_).

NO is a strong oxidant and one of the most important mediators in the regulation of endothelial cell functions, which is synthesized by three isoforms of NO synthases (NOS), that is, eNOS, iNOS, and nNOS. eNOS is constitutively expressed and there is particularly continuous NO production during physiological conditions [21]. Our results suggested that incubation with AGEs (100 mg/L) for 24 h led to an significantly increase in the NO, iNOS production and a decrease in the secretion and the mRNA expression of eNOS compared to control group in HUVECs (Figures 3–5); these effects were strikingly reversed by EM1 and EM2 pretreatment, these findings were in line with some previous reports [22, 23]. It is well recognized that NO produced by eNOS is described as “low output” pathway whereas iNOS generates NO in a “high output” manner which causes cell or organ dysfunction and apoptosis [24]. Evidence indicated that NOS isoform expression (particularly iNOS) is altered and NO is oversupplied in some pathologic conditions [25, 26]. Based on our data, we inferred that AGEs can alter NOS isoform expression by decreasing eNOS expression and stimulating iNOS oversupplied in HUVECs, resulting in overproduction of NO, which was due to enhanced iNOS expression. EMs can enhance NOS activity by up-regulating eNOS expression and decreasing iNOS production, leading to a health production and bioavailability of NO. It is indicated that EMs can attenuate the dysfunction of NOS induced by AGEs in HUVECs. EM1 showed the same effects as EM2 in our observation. A large amount of studies reported that EMs can regulate NOS expression in mammalian cells, such as human bone marrow stromal cells [27], human macrophages [28], and mice peritoneal macrophages [29]; however, to our knowledge, there was no report in HUVECs.

ET-1, a member of potent vasoconstrictor polypeptide family, has been characterized as one of the most potent endogenous vasoconstrictors [30]; the balance between NO and ET-1 is critical for the regulation of vascular tone. Our study clearly indicated that EM1 and EM2 pretreatment down-regulated the mRNA expression and the plasma concentration of ET-1, which was up-regulated by AGEs-BSA (Figure 6). We think that EMs inhibited ET-1 expression through increased NO production which is synthesised by eNOS and decreased NO production which is synthesised by iNOS, leading to a balance between ET-1 and NO, potentially contributing to endothelial function.

It was shown earlier that p38 MAPK directly phosphorylates c-Jun [31, 32]; protein phosphatases and p38 MAPK interact in various cell systems and have been implicated in the regulation of diverse cellular responses together [33]. In HUVECs, AGEs induce differentiation accompanied by activation of ERK, JNK, and p38 MAPK pathways [34]. These datum are consistent with our observation that p38 MAPK is activated by AGEs (100 mg/L); furthermore, our study showed that p38 MAPK activation was weakened in EMs pretreated groups relative to AGEs groups. The results indicated that the rescue effect of EMs on the AGEs-induced injury may be mediated, at least in part, by the p38 MAPK pathway. It is acknowledged that the research means and methods we used in the study on signal pathway seemed too monospecific, which needs to be demonstrated by various experimental methods and different angles. That is our next working direction and emphasis in future.

In conclusion, endomorphins can attenuate the HUVEC dysfunction of synthesising and secreting NO, eNOS, iNOS, ET-1 induced by AGEs and may inhibited p38 MAPK signal pathway in nucleus stimulated by AGEs. These findings have partly revealed the molecular mechanism of endomorphins on protecting HUVECs from injuries induced by AGEs and thereby may provide the pharmacologic basis for the treatment of endothelial dysfunction in diabetes.

## Figures and Tables

**Table 1:** Primers used for RT-PCR.

Gene name	Primer sequences	*T*_*m*_ (°C)	Cycles	Length (bp)	Accession number
ET-1	F: 5′-TCAGAGGAACACCTAAGACAA-3′	63.3	35	123	NM-001955.3
	R: 5′-TGCTCGGTTGTGGTCACATA-3′				
NOS	F: 5′-GCTGTCTGCATGGACCTGGA-3′	64.8	38	119	NM-000603.3
	R: 5′-TCCACGATGGTGACTTTGGCTA-3′				
*β*-actin	F: 5′-GCAAGCAGTATGACGAGT-3′	64.3	10	112	NM-001101.3
	R: 5′-CTGCGCAAGTTAGGTTTTGTC-3′				
